# Screening for germline phosphatase and tensin homolog-mutations in suspected Cowden syndrome and Cowden syndrome-like families among uterine cancer patients

**DOI:** 10.3892/ol.2015.2890

**Published:** 2015-01-23

**Authors:** GERASIMOS TZORTZATOS, CHRISTOS ARAVIDIS, ANNIKA LINDBLOM, MIRIAM MINTS, EMMA THAM

**Affiliations:** 1 Department of Women’s and Children’s Health, Division of Obstetrics and Gynecology, The Karolinska Institute, Karolinska University Hospital, Solna, Stockholm 171 76, Sweden; 2 Division of Obstetrics and Gynecology, Karolinska University Hospital, Huddinge, Stockholm 141 86, Sweden; 3 Department of Clinical Genetics, Akademiska Hospital, Uppsala University, Uppsala 751 85, Sweden; 4 Department of Clinical Genetics, The Karolinska Institute, Karolinska University Hospital, Solna, Stockholm 171 76, Sweden; 5 Department of Molecular Medicine and Surgery, The Karolinska Institute, Karolinska University Hospital, Solna, Stockholm 171 76, Sweden

**Keywords:** germline phosphatase and tensin homolog mutations, Cowden syndrome-like families, uterine cancer

## Abstract

Cowden syndrome (CS) is an autosomal dominant disorder characterized by multiple hamartomas in the breast, thyroid and endometrium, with a prevalence of 1 per 250,000. Females with CS have a 21–28% lifetime risk of developing uterine cancer. Germline mutations in the phosphatase and tensin homolog (*PTEN*) gene, a tumor suppressor gene, are responsible for 30–80% of CS cases. *PTEN* is a nine-exon gene, located on chromosome 10q23.3, which encodes the 403 amino acid PTEN protein. It negatively regulates the phosphoinositide 3-kinase/protein kinase B/mammalian target of rapamycin pathway, affecting various cellular processes and signaling pathways. The present study examined whether *PTEN* mutations are present in CS-like families with uterine cancer (UC). UC patients underwent surgery at Karolinska University Hospital, Stockholm, Sweden (2008–2012). Pedigrees were analyzed and 54 unrelated CS-like families were identified. CS-like families were defined as having at least one occurrence of uterine cancer and one of breast cancer, as well as at least one additional Cowden-associated tumor (uterine, breast, thyroid, colon or kidney cancer) in the same individual or in first-degree relatives. Genomic DNA was amplified using polymerase chain reaction, and DNA sequencing analysis of all nine exons of the *PTEN* gene was conducted. No germline *PTEN* mutations or polymorphisms were identified. Germline *PTEN* mutations are rare in CS-like families with uterine cancer, therefore, genetic screening must be restricted to patients that meet the strict National Comprehensive Cancer Network criteria. Gynecologists must be aware of the CS criteria and identify potential cases of CS in females where uterine cancer is the sentinel cancer.

## Introduction

Uterine cancer is the most common gynecological malignancy in Sweden. It accounts for 5.2% of all female malignancies, with 1431 new cases reported in 2011 (1). The majority of uterine cancer cases are endometrial carcinoma, whilst uterine sarcomas are less common, accounting for <5% of all cases.

The risk of uterine cancer increases with age, and females are typically affected between 50 and 60 years of age (2,3). The major risk factors for uterine cancer include diabetes mellitus, obesity, hypertension, polycystic ovary syndrome, anovulation, nulliparity and exposure to exogenous estrogens (2–4). There are two types of endometrial cancer: Type I and type II. Type I is preceded by endometrial hyperplasia due to estrogen stimulation. These tumors originate from endometrioid epithelium and are typically well-differentiated. Type I is associated with specific genetic alterations, including microsatellite instability due to methylation of the mismatch repair gene *MLH1*, and somatic mutations in *KRAS*, *CTNNB1*, phosphatase and tensin homolog (*PTEN*) and *PIK3CA* genes. Type II diseases are of serous or clear cell histology, and develop from the atrophic endometrium without hyperplasia. They are generally poorly differentiated and exhibit *p53* mutations, E-cadherin inactivation and HER2-amplification (3,5,6). The majority of cases of uterine cancer are sporadic; however, multiple instances of endometrial cancer within a family occurs in ~5% of all cases (7).

Cowden syndrome (CS) is an autosomal dominant disorder characterized by multiple hamartomas in the breast, thyroid, colon, kidney and endometrium, and has a worldwide prevalence of 1 in 250,000 (8). Germline mutations in *PTEN* were initially proposed to be responsible for 80% of CS cases; however, more recent studies indicate that only 30–35% of all CS cases are caused by *PTEN* mutations (9). Recent studies also indicate that the lifetime risk of developing endometrial carcinoma in patients with CS is 21–28%, with the highest risk levels occurring in individuals aged >35 years (10–12). Diagnosis is determined according to the National Comprehensive Cancer Network (NCCN) criteria (13).

The gene responsible for CS is the tumor suppressor gene *PTEN*, which is located in the 10q23.3 chromosomal region and consists of nine-exons, encoding the 403 amino acid PTEN protein. It negatively regulates the phosphoinositide 3-kinase/protein kinase B/mammalian target of rapamycin (PI3K/AKT/mTOR) pathway by the dephosphorylation of three residues of phosphatidylinositol (3,4,5)-triphosphate. This decreases the activity of kinases downstream of PI3K, including phosphoinositide dependent kinase 1 (PDK-1), AKT, mTOR and ribosomal protein s6 kinase (S6K1). In CS, the loss of activity of *PTEN* occurs after inheriting a mutated allele, followed by a second hit mutation (somatic) of the normal allele, which leads to a loss of function of the protein product and increased phosphorylation. This affects various cellular processes and signaling pathways, including cell cycle progression, metabolism, translation, growth, migration, invasion, angiogenesis and apoptosis (5,8,14).

Given the low prevalence of CS and the difficulty in identifying which patients fulfill the NCCN criteria, the present study aimed to examine whether *PTEN* mutations are present in a significant proportion of families with uterine cancer that do not meet the strict criteria but have a CS-like family hereditary pattern.

## Materials and methods

Uterine cancer patients who underwent surgery between January 2008 and March 2012 were invited to participate in the present study. All participants gave their written informed consent for data collection and genetic analysis. Those who accepted (index patients) completed a questionnaire regarding the initial diagnosis and age of onset of coincidental cancers in their family (the index patient, the first- and second-degree relatives and first cousins), including colorectal, breast, ovarian and other cancer types. At the end of the study period in 2012, all index patients were checked for relapse and/or novel primary tumors via the Swedish Cancer Registry.

Upon enrollment, all index patients provided a blood sample for DNA extraction, according to the manufacturer’s instructions (MagneSil Genomic, Large Volume System, Promega, Madison, WI, USA; Freedom EVO Tecan robot, serial no. 904004850, Tecan, Männedorf, Switzerland), at the Department of Clinical Genetics, Karolinska University Hospital (Stockholm, Sweden), and their histological results were obtained. Telephone interviews were conducted to acquire information with regard to cancer diagnosis in relatives. For all relatives with cancer, the current age or age at mortality, type of cancer and age at diagnosis was recorded. Histological verification of cancer diagnoses in relatives was obtained from the Swedish Cancer Registry, patients’ medical records and/or their death certificates. All data was acquired following the written consent of the affected relative (or, if deceased, from their closest living relative).

**Figure 1:**
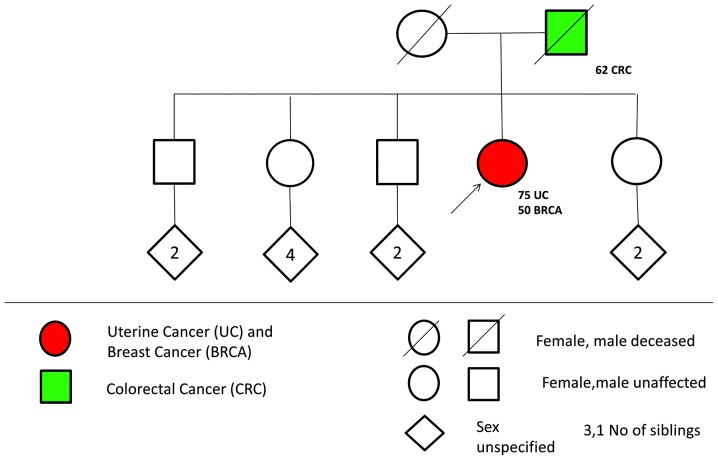
Pedigree of a Cowden syndrome-like family.

Pedigrees were constructed for each index patient based on the information provided in the questionnaires and the telephone interview. All pedigrees were evaluated for the possible presence of CS using the NCCN guidelines. CS-like families were defined by the presence of at least one uterine cancer and one breast cancer, as well as at least one additional Cowden-associated tumor (uterine, breast, thyroid, colon or kidney cancer), in the same individual or first-degree relatives (Fig. 1).

Genomic DNA was subjected to touchdown polymerase chain reaction (PCR) for the amplification of all nine exons of the *PTEN* gene and their flanking intronic regions. Firstly, seven cycles of amplification with denaturation at 95°C for 30 sec, an annealing gradient for 45 sec (Table I) and extension at 72°C for 45 sec was performed. Next, a further 30 cycles of amplification were performed with denaturation at 95°C for 30 sec, annealing at 55–56°C (Table I) for 45 sec and extension at 72°C for 45 sec. The final extension step was performed as follows: 72°C for 10 min followed by cooling of the product overnight at 4°C. PCR amplification was performed in a final volume of 25 μl, using AmpliTaq Gold DNA Polymerase (Roche Molecular Diagnostics, Pleasanton, CA, USA), 10 μM final primer concentration (Table 1) and 50 ng template DNA from each patient, in a 2720 Thermal Cycler (Applied Biosystems, Foster City, CA, USA) or a DNA Engine Tetrad 2 Peltier Thermal Cycler (Bio-Rad, Hercules, CA, USA). PCR products were purified with ExoSAP-IT (USB Affymetrix, Santa Clara, CA, USA) and subsequently sequenced by Sanger sequencing using a 48-capillary 3730xl DNA Analyzer (Applied Biosystems). The resulting sequences were analyzed using Seqscape software version 2.7 (Invitrogen Life Technologies, Carlsbad, CA, USA), with the reference sequence NM 000314.4.

The study was approved by the Ethics Committee of Karolinska Institute/Karolinska University Hospital (Stockholm, Sweden).

## Results and Discussion

A cohort of 54 unrelated patients with uterine cancer and CS-like phenotype was identified. The characteristics of the patients are shown in Table II. None of the individuals included in the present study fulfilled the CS diagnostic criteria. No germline mutations or polymorphisms were identified in the coding region of *PTEN* in the population of CS-like families.

As CS is extremely rare, only small-scale studies have searched for *PTEN* mutations in families with CS or CS-like phenotype (5,15–22), and none of these studies investigated families with uterine cancer. Patients with uterine cancer and CS-like family history were referred to our oncogenetic clinic, however, the value of testing for mutations in *PTEN* in this patient category is unclear. Marsh *et al* (15) reported one family with a *PTEN* mutation among 64 CS-like families with breast and thyroid cancer, suggesting that certain CS-like families may have CS without fulfilling the strict NCCN criteria (11). However, the majority of studies have not identified *PTEN* mutations in CS-like families. These studies included families with breast and thyroid cancer (16,17), hereditary breast cancer (18–20), breast and central nervous system cancer (21), consecutive cases of thyroid cancer (22) or consecutive endometrial cancer (5). The present study indicates that *PTEN* mutations are not the cause of cancer in CS-like families with uterine cancer.

The two major flaws of the current study are the small number of patients included in the sample, and the lack of detailed phenotypic evaluation of the patients in order to obtain information on head circumference, and on non-cancer phenotypes. Macrocephaly is one of the required major diagnostic criteria for Cowden syndrome and therefore measurements of head circumference are important. However, information on head circumference and discrete mucocutaneous lesions is often lacking on patients with endometrial cancer and a family history of Cowden-associated tumors, who are referred for clinical genetic testing of *PTEN* in our clinic. The study included the DNA sequencing of the coding region of *PTEN*, where 90% of all CS-mutations are detected. However, deletions/duplications were not investigated, as larger deletions in *PTEN* have been demonstrated in only 1% of all cases (23). The promoter region of *PTEN*, where mutations have been observed in 10% of all patients with classic CS (23), was also excluded. Despite these limitations, the results indicate that *PTEN* mutations are rare in patients with uterine cancer and other CS-related tumors, and clinical testing of *PTEN* is thus not indicated if the NCCN criteria are not met.

Notably, *PTEN-*negative CS and CS-like patients have been demonstrated to exhibit hypermethylation of the bidirectional promoter that regulates *PTEN* and *KILLIN*, which is associated with an increased risk of breast and kidney cancer (24). Rare cases also demonstrate mutations in succinate dehydrogenase complex (*SDH*) subunit D, *SDHB*, *PI3KCA*, *AKT1* and *RAS* GTPase activating protein genes, suggesting that *PTEN-*negative cases of CS are genetically heterogeneous (25–27). Thus, diagnosis of CS and CS-like patients may be improved by utilizing a targeted gene panel including the aforementioned genes, in combination with a methylation analysis. This was beyond of the scope of the present study, and may be a perspective for future studies.

In conclusion, germline *PTEN* mutations are rare in a population of CS-like families with uterine cancer. The high cost of routine screening for *PTEN* mutations among endometrial cancer patients is not justified at an oncogenetic clinic, and must be restricted to patients that meet the strict Cowden criteria. Gynecologists must be aware of the CS criteria in order to identify potential cases of CS in females where uterine cancer is the sentinel cancer.

## Figures and Tables

**Table I:** The primer pairs for every amplicon (forward and reverse), their corresponding melting and annealing temperatures, GC% and the polymerase chain reaction product size.

*PTEN* exon	Forward primer 5′-3′	Tm, °C	GC %	Reverse primer 5′-3′	Tm, °C	GC %	Amplicon size, bp	Initial temp, °C	Final temp, °C
Exon 1	TTCCATCCTGCAGA AGAAGC	60.5	50	CATCCGTCTACTC CCACGTT	60.0	55	230	62	55
Exon 2	TGGGGAAA(A/G)C TTTCTTTTCA	57.3	35	CATCACAAAGTAT CTTTTTCTGTGG	59.1	36	293	62	55
Exon 3	CCATAGAAGGGGT ATTTGTTGG	59.6	45.5	CAATGCTCTTGGA CTTCTTGA	58.1	42.9	364	62	55
Exon 4	AAAGATTCAGGCA ATGTTTGTT	57.8	31.8	TCTCACTCGATAA TCTGGATGAC	58.3	43.5	235	62	56
Exon 5	TGCAACATTTCTAA AGTTACCTACTTG	59.3	33.3	GAAACCCAAAAT CTGTTTTCCA	60.2	36.4	398	62	56
Exon 6	GGCTACGACCCAG TTACCAT	58.9	55	CCTGCATAAATTT CAAATGTGG	59.4	36.4	347	61	55
Exon 7	AAAATCGTTTTTGA CAGTTTGACA	60.0	29.2	CACCAATGCCAG AGTAAGCA	59.9	50	379	61	55
Exon 8	TGTTTAACATAGGT GACAGATTTTCTT	58.7	29.6	CTCCTAGAATTAA ACACACATCACA	57.5	36	396	61	55
Exon 9	TGTTCATCTGCAAA ATGGAAT	58.1	33.3	CTGGTAATCTGAC ACAATGTCCT	58.1	43.5	399	61	55

PTEN, phosphatase and tensin homolog; bp, base pairs; Tm, melting temperature.

**Table II:** Characteristics of the index patients (n=54).

Characteristics	n/total	%
Age at diagnosis, years		
Median (range)	75 (45–87)	
Histology		
Endometrioid	46/54	85.2
Serous or mixed	4/54	7.4
Clear cell	3/54	5.6
Sarcoma	1/54	1.8
Hyperplasia with atypia	0/54	0
FIGO stage		
1A	35/54	64.8
1B	10/54	18.5
2	5/54	9.3
3A	1/54	1.8
3B	0/54	0
3C	0/54	0
4	0/54	0
4B	3/54	5.6
Grade		
1	21/54	38.9
2	22/54	40.7
3	11/54	20.4
Depth of myometrial invasion		
None	7/54	12.9
<50%	31/54	57.4
≥50%	14/54	26
Through the serosa	2/54	3.7
Relapse	0	
Other cancer in the same individual	19/54	35.2
Breast cancer	15/19	78.9
Colorectal cancer	1/19	5.3
Combined	3/19	15.8
Other cancer in the family (FDRs)^a^	52	
Breast cancer	27/52	51.9
Colorectal cancer	12/52	3.8
Endometrial cancer	1/52	1.9
Combined^b^	12/52	3.8

a Two had no relatives with other cancers, but had other cancers themselves and thus qualified as CS-like family;

b at least one FDR with more >1 different cancers.

FIGO, International Federation of Obstetrics and Gynecology; FDR, first-degree relative.
